# Circulating Fractalkine Levels Predict the Development of the Metabolic Syndrome

**DOI:** 10.1155/2014/715148

**Published:** 2014-04-30

**Authors:** Yin Xueyao, Zhang Saifei, Yu Dan, Pan Qianqian, Dong Xuehong, Zhou Jiaqiang, Zheng Fenping, Li Hong

**Affiliations:** Department of Endocrinology, The Affiliated Sir Run Run Shaw Hospital, School of Medicine, Zhejiang University, 3 East Qingchun Road, Hangzhou 310016, China

## Abstract

The fractalkine/CX3CR1 axis plays an important role in regulating glucose and lipid metabolism. However, the role of fractalkine in metabolic disorders remains to be fully elucidated. We selected 887 Chinese (40–65 years old) at baseline, with a subgroup of 459 participants examined again 2 years later. The relationship of serum fractalkine levels with the metabolic syndrome (MetS) and its components was investigated. At baseline, participants with MetS had higher fractalkine concentrations than their counterparts without MetS (*P* < 0.001). At the 2-year follow-up, participants in the highest quartile of baseline fractalkine exhibited higher values for body mass index, waist circumference, waist-to-hip ratio, body fat percentage, glucose, insulin, total cholesterol, triglycerides (TG), and homeostasis model assessment of insulin resistance (HOMA-IR) and lower value for high density lipoprotein-cholesterol (HDL-c) (all *P* < 0.05). Among 390 participants without MetS at baseline, 45 developed it at year 2. Even after multiple adjustments for visceral adipose tissue area, HOMA-IR, C-reactive protein (CRP), or TG and HDL-c, baseline fractalkine predicted the development of MetS (OR = 7.18, 95%CI: 2.28–18.59). In conclusion, circulating fractalkine predicts the development of the MetS independently of central obesity, CRP, insulin resistance, and dyslipidemia.

## 1. Introduction

Metabolic syndrome (MetS) comprises a group of conditions, including central obesity, dyslipidemia elevated blood pressure (BP), and abnormal glucose metabolism. It is associated with increased risk of type 2 diabetes (T2DM) and cardiovascular disease (CVD). The prevalence of MetS has been increasing dramatically in China during the past decade, accompanied by the rapid economic growth and adoption of a sedentary lifestyle [1–3]. Pathophysiologically, MetS is characterized by chronic low-grade inflammatory responses which are associated with abnormal levels of cytokines and other inflammatory signaling markers [4–6].

Fractalkine (CX3CL1), the only known member of the CX3C class of chemokines, is known to convey its signals through a single G-protein-coupled receptor, CX3CR1, thereby promoting leukocyte activation and survival [7]. Fractalkine expression has been detected in activated or stressed endothelial, smooth muscle cells, skeletal muscle, macrophages, neurons, hepatocytes [8–12], and adipocytes [13]. It is characterized as a structurally unique chemokine, with both membrane-bound and soluble forms that act, respectively, to promote cell-to-cell adhesion of circulating leukocyte or as a classical chemoattractant of monocytes and lymphocytes [9, 14–16]. The soluble fractalkine is generated by cleavage of the membrane-bound form by two peptidases, ADAM10 and ADAM17 [17, 18].

Patients with unstable angina pectoris and plaque rupture [19] or CVD [8] show strongly enhanced activation of the fractalkine/CX3CR1 axis, and this signal has been implicated in the development of these pathogenic processes. A recent study reported that inflammation upregulates fractalkine, particularly in the adipose tissue of obese individuals and T2DM patients [13]. A putative explanation for the association between fractalkine and MetS was published recently [20], but the evidence of such a relationship remains scarce. Therefore, this study was designed to investigate the relationship between baseline serum fractalkine and the development of MetS using a group of middle-aged Chinese adults. In addition, the fractalkine-MetS association was evaluated to determine any potential dependence upon well-established risk factors of MetS, such as central obesity, C-reactive protein (CRP), insulin resistance, and dyslipidemia.

## 2. Methods and Procedures

### 2.1. Study Design

This population-based cross-sectional survey was conducted from March to May 2010 in the Caihe community of Hangzhou, Zhejiang province, China. A total of 887 eligible Han Chinese participants, aged 40–65 years, were recruited in the baseline study. None of the participants had a previous diagnosis of diabetes, moderate to severe hypertension (resting BP > 170/100 mmHg), other CVD, chronic renal disease, acute infectious disease or chronic inflammatory disease, endocrine disease, cancer, or treatment with lipid-lowering drugs. During the 2-year follow-up period, 428 participants dropped out because of death (*n* = 8), loss of contact (*n* = 143), or withdrawal from the study (*n* = 277). At the end of the study, 459 participants were followed up. The study protocol was approved by the Ethics Committee of Sir Run Run Shaw Hospital and conducted in accordance with the Declaration of Helsinki. Written informed consent was obtained from all participants. Face-to-face interviews were conducted by trained medical staff using a standardized questionnaire to collect participant demographic data and to obtain baseline lifestyle and health status information.

### 2.2. Laboratory Measurements

Participants visited local community health care centers between 7 and 8 am following an overnight fast. Venous blood samples were collected at 0 and 2 hours following a 75-g oral glucose tolerance test (OGTT). Blood samples obtained for laboratory testing were immediately centrifuged, and the serum was stored at −80°C. Serum glucose concentrations, triglyceride (TG), total cholesterol (TC), low density lipoprotein-cholesterol (LDL-c), high density lipoprotein-cholesterol (HDL-c), and CRP were assayed with an autoanalyzer (Aeroset, Chicago, IL, USA). Glycosylated hemoglobin A_1_c (HbA_1_c) was measured by ion-exchange high-performance liquid chromatography (Hemoglobin Testing System; Bio-Rad, Hercules, CA, USA). Serum insulin levels were measured by a radioimmunoassay using an insulin detection kit (Beijing North Institute of Biological Technology, China). Homeostatic model assessment of insulin resistance (HOMA-IR) was calculated using the following formula: [fasting serum insulin (FINS; mU/L) × fasting serum glucose (FPG; mmol/L)/22.5] [21]. Fractalkine concentration was determined with a commercially available enzyme-linked immunosorbent assay (R&D Systems, Minneapolis, MN, USA). All assay procedures followed the manufacturer's instructions. Intra- and interassay coefficient of variation were 1.7% to 4.3% and 3.5% to 7.9%, respectively.

### 2.3. Anthropometric Measurement

Body mass index (BMI) was calculated by dividing body weight by height squared (kg/m^2^). Waist circumference (WC) was measured at the midpoint between the lower border of the rib cage and the iliac crest. Hip circumference was measured at the widest point of the hips, and the waist-to-hip ratio (WHR) was calculated and recorded for each patient. Both measurements were taken while the patient was standing. Body fat percentage (Fat%) was measured by bioelectrical impedance analysis (TBF-300, Tanita Co., Tokyo, Japan). Systolic blood pressure (SBP) and diastolic blood pressure (DBP) were measured in triplicate using a mercury sphygmomanometer, and the average of the three measurements was recorded.

Abdominal adipose tissue was measured using a whole-body imaging system (SMT-100, Shimadzu Co., Kyoto, Japan) with TR-500 and TE-200 of SE. Magnetic resonance imaging (MRI) was performed at the umbilical level with the participant in a supine position; abdominal visceral adipose tissue area (VFA) and abdominal subcutaneous adipose tissue area (SFA) were calculated with the accompanying software.

### 2.4. Definition of MetS

MetS was defined according to criteria established by the Joint Committee for Developing Chinese Guidelines on Prevention and Treatment of Dyslipidemia in Adults (JCDCG) [22]. Individuals with three or more of the following abnormalities were considered as having MetS: central obesity (WC > 90 cm for men and >85 cm for women); hypertriglyceridemia (≥1.70 mmol/L); low HDL-C (<1.04 mmol/L); elevated BP (≥130/85 mmHg or current treatment for hypertension); and hyperglycemia (FPG ≥ 6.1 mmol/L or 2 h postprandial glucose (2 h PG) ≥7.8 mmol/L).

### 2.5. Statistical Analyses

Normally distributed variables were expressed as mean ± standard deviation (SD); variables with a skewed distribution, including fractalkine, insulin, glucose, HOMA-IR, TC, TG, CRP, SFA, and VFA, underwent a lg(x) transformation to achieve a normal distribution and were reported as median value (interquartile range) [M(IQR)]. Categorical variables were expressed as frequency and percentage. Fractalkine levels of the 459 participants with 2-year follow-up data were grouped into quartiles to simplify the interpretation of the results of subsequent analyses. The chi-squared test was used to compare categorical variables between groups. For continuous variables,* t*-test was used to compare between 2 groups, and ANOVA test was used for comparison of multiple groups. Bivariate correlation analyses between fractalkine and the metabolic parameters were performed using Pearson's correlation analysis. The adjusted odds ratios (ORs) for the development of MetS at year 2 according to the baseline fractalkine quartiles were calculated in multivariate logistic regression models. Potential confounders, including age, sex, and lifestyle factors, were carefully controlled. Potential interactions between VFA, CRP, HOMA-IR, TG, HDL-c, and fractalkine were also examined. All statistical analyses were performed with SPSS 20.0 (IBM, Armonk, NY, USA) and considered statistically significant when the 2-sided *P* value was <0.05.

## 3. Results

The baseline characteristics for all participants are shown in Table 1. The mean (±SD) age was 56.90 (±7.28) years, and 39.5% of the participants were male. Among the participants, 14.2% had MetS. The median (range) for serum fractalkine was 0.44 (0.28–0.65) ng/mL for males and 0.40 (0.25–0.62) ng/mL for females (*P* = 0.379). As expected, participants with MetS at baseline had a greater number of adverse risk factors than participants without MetS, including higher BMI, WHR, Fat%, insulin, HOMA-IR, HbA_1_c, TC, CRP, SFA, VFA, and MetS defining parameters (Table 1, *P* < 0.05 for all parameters). In addition, the fractalkine concentration was significantly higher in participants with the MetS (Table 1, *P* < 0.001).

Serum fractalkine concentration was positively correlated with BMI, WC, WHR, Fat%, BP, blood glucose, insulin, HOMA-IR, TC, TG, SFA, and VFA but negatively correlated with serum HDL-c after adjustment for age, sex, education, smoking, and drinking at baseline (Table 2). Of all the metabolic parameters, fractalkine showed the strongest correlation with VFA (*r* = 0.28, *P* < 0.001).

**Figure 1:**
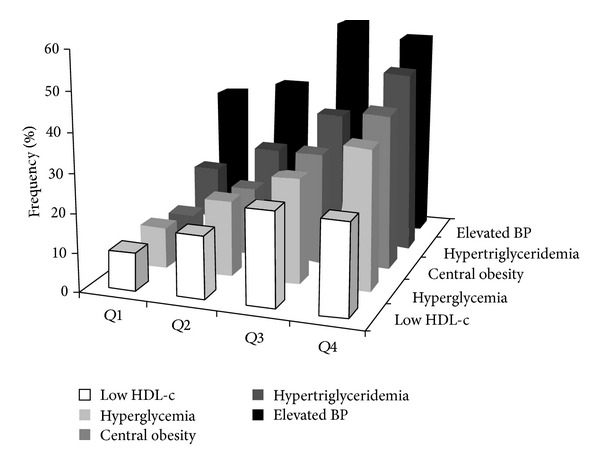
The frequency of each MetS component plotted according to baseline fractalkine quartiles at year 2.

**Figure 2:**
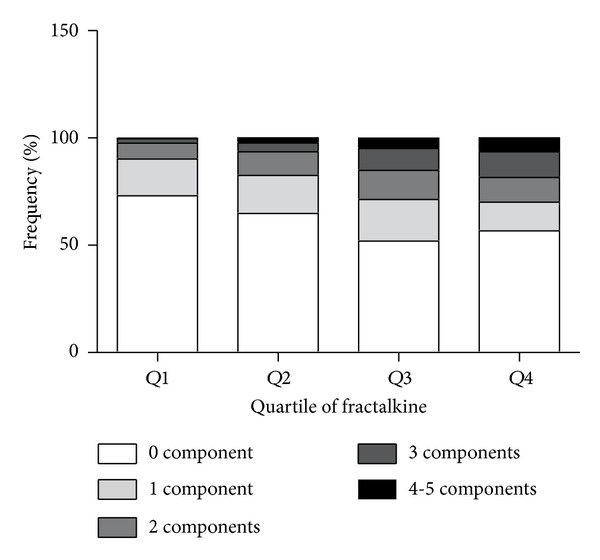
The number of MetS components plotted according to baseline fractalkine quartiles at year 2.

After the 2-year follow-up, 459 participants were completely investigated. There was no significant difference in baseline characteristics between these subjects and the general participants (see Table 1 in Supplementary Material available online at http://dx.doi.org/10.1155/2014/715148). Among them, 399 participants did not have MetS at baseline. Participants in the higher fractalkine quartiles at baseline exhibited higher levels for BMI, WC, WHR, Fat%, blood glucose, insulin, HOMA-IR, TC, and TG (all *P* < 0.05) than participants in the lower quartile after 2 years. In addition, participants with higher fractalkine levels had lower HDL-c levels (*P* = 0.035). The prevalence of MetS and each component at year 2 increased along with the elevation of baseline fractalkine concentration (Table 3 and Figure 1). As presented in Table 4, the baseline age-, sex-, education-, smoking-, and drinking-adjusted fractalkine was found to have significant positive correlations with multiple adverse metabolic parameters at year 2, including high BMI, WC, WHR, Fat%, blood glucose, insulin, HOMA-IR, HbA_1_c, and TG. On the other hand, a significant negative correlation was found between baseline fractalkine and HDL-c level at year 2. In addition, the number of MetS components, indicated as ≤1, 2, 3, and ≥4, increased gradually across the baseline fractalkine concentration at the 2-year follow-up (Figure 2).

Among 399 participants who did not have MetS at baseline, 45 had developed MetS at year 2. Nine of 399 participants were on statins at year 2 but did not have pretreatment lipid profiles for accurate classification of the MetS status and hence were excluded from year 2 analysis. The baseline fractalkine concentrations were significantly higher in participants who had progressed to MetS by year 2 than in participants without MetS [0.51 (0.36–0.68) versus 0.40 (0.24–0.58), *P* < 0.001]. In the multiple stepwise logistic regression analysis, participants in the higher quartiles for fractalkine had higher OR for the development of MetS and its components by year 2.Table 5 (model 2) showed that, compared with the lowest quartile of fractalkine concentration, the ORs in the highest quartile were 7.18 (95% CI: 2.28–18.59) for MetS, 4.83 (95% CI: 2.09–11.19) for central obesity, 1.03 (95% CI: 0.55–1.93) for elevated BP, 3.61 (95% CI: 1.63–8.02) for hyperglycemia, 2.63 (95% CI: 1.30–5.34) for hypertriglyceridemia, and 1.59 (95% CI: 0.60–4.24) for low HDL-c. Further adjustment for VFA (model 3), VFA and CRP (model 4), HOMA-IR (model 5), or TG and HDL-c (model 6) only slightly reduced the magnitude of the association of baseline fractalkine with the development of MetS (OR = 5.31, 95% CI: 1.65–14.09 for model 3, OR = 5.17, 95% CI: 1.60–13.74 for model 4, OR = 5.73, 95% CI: 1.79–14.34 for model 5, and OR = 5.94, 95% CI: 1.85–15.09 for model 6). These results suggest that the association between fractalkine and the development of MetS is independent of central obesity, CRP, insulin resistance, and dyslipidemia. In addition, circulating fractalkine concentration was significantly associated with the development of each MetS component (Table 5). The associations of fractalkine with hyperglycemia were particularly strong and independent of  VFA; the association between fractalkine and the other MetS components was largely explained by central obesity.

## 4. Discussion

Altered circulating cytokine levels can be used as early abnormal markers and may contribute to MetS development. This study addressed the relationship between fractalkine and the development of MetS in a 2-year prospective study. We found that elevated serum fractalkine concentrations were significantly correlated with the development of MetS. And the MetS severity at the 2-year follow-up defined as the number of MetS components increased along with the elevation of baseline fractalkine concentration. Central obesity, insulin resistance, inflammatory marker (CRP), and dyslipidemia are well-established risk factors of MetS [23–25]. However, in this study, adjustments for VFA, CRP, HOMA-IR, or TG and HDL-c and other potential confounders yielded only minor reductions in the risk of MetS development across fractalkine quartiles. Thus, the observed association between fractalkine concentrations and development of MetS cannot be attributed mainly to central obesity, CRP, insulin resistance, or dyslipidemia.

In this study, body composition was assessed not only by BMI, WC, WHR, and Fat%, but also by SFA and VFA. Body fat distribution, especially visceral fat accumulation, is more strongly correlated with obesity-related metabolic disorders than the overall amount of body fat [26, 27]. Compared with subcutaneous fat, visceral adipose tissue is known to have more extensive inflammatory leukocyte infiltration [28] and adipocytokines content [29]. Shah et al. [13] reported that fractalkine levels in subcutaneous adipose were significantly higher in obese individuals compared to their lean counterparts and that fractalkine concentrations were more strongly correlated with visceral than subcutaneous adiposity. However, they did not report an observation of increased serum fractalkine concentrations in the obese participants. Recent studies in 3306 middle-aged UK women and in a group of obese Mexican-American children both showed higher fractalkine levels in obese participants with MetS than in nonobese participants. However, the differences did not reach statistical significance [30, 31]. Our analysis showed a positive correlation between serum fractalkine levels and BMI, WC, WHR, Fat%, SFA, and VFA. The discrepancies between those studies and our results might be explained by differences in study design and in the methods of selecting the study participants.

There are also differences in the available study data describing the association between fractalkine levels and hyperglycemia. Shah et al. [13] reported that serum fractalkine concentrations were significantly higher in 281 patients with T2DM than in 274 nondiabetic participants. Another study using a cohort of middle-aged UK women showed that higher fractalkine levels were correlated with elevated insulin levels [30]. Our data from both cross-sectional and prospective studies also suggest that serum fractalkine is positively associated with glucose and insulin. However, in another study of CVD patients with and without T2DM or with and without MetS, no differences in circulating fractalkine concentration or expression of CX3CR1 were observed [32]. The lack of correlation between fractalkine levels and diabetes has also been reported by others [8, 33, 34]. Accumulating evidence, mainly from cell culture and animal studies, suggests that high glucose concentrations, similar to those seen in type 2 diabetes, promote the expression of fractalkine by smooth muscle cells and endothelial cells* in vitro*, which may then enhance monocyte adhesion and potentially promote atherogenesis [35, 36].

Relationships between circulating fractalkine concentrations and the lipoprotein-lipid profile have been observed in some studies. Franco et al. [30] reported that increased fractalkine levels correlated with elevated levels of Apo-B and LDL-c. Statin therapy can significantly reduce the expression of fractalkine and CX3CR1 [33]. In the present study, we found significant correlations between circulating fractalkine and TG and HDL-c at baseline and at the 2-year follow-up. And the fractalkine concentrations were associated with the development of hypertriglyceridemia, however, which was largely mediated by VFA or HOMA-IR. Therefore, it may also be possible that relationships reported in other cross-sectional studies between the lipid profile and fractalkine levels were not causal but largely explained by the concomitant variation in central obesity or insulin resistance.

Recent studies have shown that inflammatory cytokines, such as TNF-*α*, IFN-*γ*, and IL-1*β*, may upregulate membrane-bound fractalkine expression and the release of functional, soluble fractalkine from the bound form [9, 37, 38]. Notably, in our study, we did not observe a significant correlation between fractalkine and CRP. And the effect of CRP on the fractalkine-MetS association was rather minor. Together, this data suggested that fractalkine might provide incremental value in MetS prediction beyond current approaches. Further studies are required to determine whether an increase in circulating fractalkine is merely a reflection of obesity-related inflammation or the result of specific regulation by common mediators in adipocytes.

There are some limitations in the present study. First, because of the relatively short follow-up time of 2 years, only a small number of participants developed MetS. Whether serum fractalkine levels can be useful in predicting MetS has to be confirmed in studies involving larger populations with different genetic and environmental backgrounds. Secondly, although participants with a higher baseline serum fractalkine level present a higher risk of developing MetS, we did not have sufficient data on cardiovascular end points to investigate whether this would translate into a greater risk of cardiovascular mortality or morbidity. Thirdly, the dietary intake and work-related physical activity were not assessed in our study. Thus, the data are subject to potential under- or overestimation.

In conclusion, in this population-based middle-aged Chinese cohort, we have shown that serum fractalkine levels could predict the development of the MetS. Our findings suggest that fractalkine plays a potential role in the pathogenesis of MetS that is independent of its relationship with central obesity as reflected by VFA, insulin resistance as reflected by HOMA-IR, systemic inflammation as reflected by CRP, and dyslipidemia as reflected by TG and HDL-c. Further studies are required to investigate the efficacy of fractalkine as a biomarker or intervention target for MetS.

## Supplementary Material

Supplementary table 1. Baseline Characteristics of Participants Completely Investigated According to the Presence or Absence of the Mets (n=459).

## Figures and Tables

**Table 1:** Baseline characteristics of participants according to the presence or absence of the MetS (*n* = 887).

Variables	Total	Metabolic syndrome absent	Metabolic syndrome	*P* for trend
*n* (case/control)	887	761	126	
Fractalkine (ng/mL)	0.41 (0.25–0.64)	0.40 (0.23–0.59)	0.65 (0.48–0.74)	<0.001
Age (years)	56.90 ± 7.28	56.72 ± 7.34	57.96 ± 6.81	0.077
Male, *n* (%)	350 (39.5)	271 (35.6)	79 (62.7)	<0.001
Education level, *n* (%)				0.668
Less than high school	80 (9.0)	68 (8.9)	12 (9.5)	
High school	674 (76.0)	582 (76.5)	92 (73.0)	
More than high school	133 (15.0)	111 (14.6)	22 (17.5)	
Current smoker, *n* (%)	218 (24.6)	162 (21.3)	56 (44.4)	<0.001
Alcohol drinker, *n* (%)	187 (21.1)	146 (19.2)	41 (32.5)	0.001
BMI (kg/m^2^)	23.46 ± 2.93	23.02 ± 2.68	26.13 ± 2.96	<0.001
WC (cm)	78.44 ± 9.06	76.76 ± 8.18	88.62 ± 7.34	<0.001
WHR	0.87 ± 0.07	0.86 ± 0.07	0.94 ± 0.06	<0.001
Fat% (%)	29.14 ± 7.05	28.57 ± 6.77	32.55 ± 7.75	<0.001
SBP (mm Hg)	124.04 ± 16.57	122.15 ± 16.02	135.41 ± 15.32	<0.001
DBP (mm Hg)	81.02 ± 9.92	79.90 ± 9.60	87.71 ± 9.20	<0.001
FPG (mmol/L)	4.83 (4.50–5.22)	4.78 (4.44–5.17)	5.17 (4.78–6.11)	<0.001
2 h PG (mmol/L)	5.33 (4.50–6.50)	4.72 (4.11–5.61)	7.33 (5.22–9.44)	<0.001
FINS (*μ*U/mL)	10.64 (8.19–13.86)	10.09 (8.02–12.92)	14.95 (11.40–18.66)	<0.001
2 h INS (*μ*U/mL)	56.45 (37.17–86.06)	53.08 (35.61–80.24)	86.44 (52.88–155.52)	<0.001
HOMA-IR	2.27 (1.72–3.09)	2.11 (1.65–2.83)	3.60 (2.62–5.12)	<0.001
HbA_1_c (%)	5.64 ± 0.60	5.59 ± 0.54	5.98 ± 0.83	<0.001
TC (mmol/L)	5.57 (4.90–6.24)	5.54 (4.87–6.22)	5.75 (5.21–6.42)	0.017
LDL-c (mmol/L)	2.42 ± 0.59	2.42 ± 0.58	2.44 ± 0.65	0.664
HDL-c (mmol/L)	1.46 ± 0.37	1.52 ± 0.36	1.12 ± 0.24	<0.001
TG (mmol/L)	1.29 (0.95–1.82)	1.22 (0.89–1.59)	2.38 (1.84–3.39)	<0.001
CRP (mg/dL)	0.63 (0.28–1.59)	0.60 (0.27–1.41)	1.77 (0.79–4.38)	0.003
SFA (cm^2^)	154.6 (118.90–203.90)	152.90 (115.65–201.50)	176.40 (137.58–211.83)	<0.001
VFA (cm^2^)	69.41 (45.90–110.50)	63.39 (41.77–97.76)	127.35 (102.40–163.18)	<0.001
Central obesity (%)	122 (13.8)	53 (7.0)	69 (55.2)	<0.001
Elevated BP (%)	467 (52.6)	353 (46.4)	114 (90.5)	<0.001
Hyperglycemia (%)	134 (15.1)	71 (9.3)	63 (50.0)	<0.001
Hypertriglyceridemia (%)	266 (30.0)	152 (20.0)	114 (90.5)	<0.001
Low HDL-c (%)	83 (9.4)	25 (3.3)	58 (46.0)	<0.001

Variables with normal distributions are presented as mean ± SD; skewed variables are presented as the median value (interquartile range) [M (IQR)]. The chi-squared test was used for categorical values and *t*-test for continuous. *P* for trend depicts the significance in the difference of the mean values between participants with and without metabolic syndrome. MetS: metabolic syndrome; BMI: body mass index; WC: waist circumference; WHR: waist-hip ratio; Fat%: body fat percentage; SBP: systolic blood pressure; DBP: diastolic blood pressure; FPG: fasting serum glucose; 2 h PG: 2-hour postprandial glucose; FINS: fasting insulin; 2 h INS: 2-hour insulin; HOMA-IR: homeostatic model assessment of insulin resistance; HbA_1_c: glycosylated hemoglobin A_1_c; TC: total cholesterol; LDL-c: low density lipoprotein-cholesterol; HDL-c: high density lipoprotein-cholesterol; TG: triglyceride; CRP: C-reactive protein; SFA: subcutaneous adipose tissue area; and VFA: visceral adipose tissue area.

**Table 2:** Correlations between serum fractalkine and metabolic parameters at baseline (*n* = 887).

	Unadjusted		Age-, sex-, education-, smoking-, and drinking-adjusted	
	r	P value	*r*	*P* value
BMI	0.24	<0.001	0.16	0.001
WC	0.29	<0.001	0.23	<0.001
WHR	0.26	<0.001	0.20	<0.001
Fat%	0.23	<0.001	0.23	<0.001
SBP	0.23	<0.001	0.12	0.019
DBP	0.19	<0.001	0.12	0.013
FPG^a^	0.19	<0.001	0.14	0.006
2 h PG^a^	0.25	<0.001	0.24	<0.001
FINS^a^	0.21	<0.001	0.17	0.001
2 h insulin^a^	0.24	<0.001	0.24	<0.001
HOMA-IR^a^	0.25	<0.001	0.20	<0.001
HbA_1_c	0.08	0.020	0.10	0.053
TC^a^	0.16	<0.001	0.10	0.040
LDL-c	0.17	<0.001	0.18	0.058
HDL-c	−0.14	<0.001	−0.15	<0.001
TG^a^	0.24	<0.001	0.20	<0.001
CRP^a^	0.10	0.030	0.09	0.062
SFA^a^	0.20	<0.001	0.20	<0.001
VFA^a^	0.32	<0.001	0.28	<0.001

Abbreviations as in Table 1.

Coefficients were performed using Pearson's correlation analysis.

^a^lg(x) transformation was performed because of a skewed distribution.

**Table 3:** Association of baseline fractalkine concentrations with metabolic parameters at year 2 (*n* = 459).

Variables	Q1	Q2	Q3	Q4	*P* for trend
	≤0.28 ng/mL	0.28–0.43 ng/mL	0.43–0.64 ng/mL	>0.64 ng/mL	
*n* (case/control)	115	114	115	115	
Fractalkine (ng/mL)	0.18 (0.10–0.23)	0.35 (0.30–0.40)	0.51 (0.48–0.57)	0.71 (0.66–0.78)	<0.001
Age (years)	57.92 ± 6.58	57.75 ± 6.80	60.57 ± 6.01	60.73 ± 6.44	<0.001
Male, *n* (%)	39 (35.4)	42 (37.8)	44 (38.9)	45 (39.8)	0.855
Education					0.992
Less than high school	11 (9.7)	11 (9.9)	11 (9.7)	13 (11.5)	
High school	86 (76.1)	83 (74.8)	88 (77.9)	83 (73.5)	
More than high school	16 (14.2)	17 (15.3)	14 (12.4)	17 (15.0)	
Current smoker, *n* (%)	24 (21.2)	24 (21.6)	22 (19.5)	29 (25.7)	0.717
Alcohol drinker, *n* (%)	15 (13.3)	24 (21.6)	25 (22.1)	29 (25.7)	0.127
MetS, *n* (%)	6 (6.6)	14 (12.6)	31 (27.4)	40 (35.4)	<0.001
BMI (kg/m^2^)	22.84 ± 2.50	24.04 ± 2.64	24.77 ± 2.84	24.83 ± 2.86	0.001
WC (cm)	75.71 ± 8.31	80.61 ± 7.67	83.69 ± 9.13	85.35 ± 9.25	<0.001
WHR	0.84 ± 0.07	0.88 ± 0.06	0.89 ± 0.07	0.91 ± 0.08	<0.001
Fat% (%)	27.92 ± 9.71	29.52 ± 6.82	30.75 ± 6.29	31.51 ± 8.75	0.006
SBP (mm Hg)	117.05 ± 14.34	117.32 ± 11.78	121.11 ± 13.97	120.71 ± 14.81	0.117
DBP (mm Hg)	70.24 ± 9.78	71.19 ± 8.39	73.85 ± 9.69	72.77 ± 9.54	0.069
FPG (mmol/L)	5.00 (4.70–5.20)	5.10 (4.90–5.45)	5.10 (4.80–5.50)	5.30 (5.00–5.85)	<0.001
2 h PG (mmol/L)	5.50 (4.30–6.50)	5.60 (4.65–6.60)	5.80 (5.00–7.00)	5.90 (5.10–7.95)	<0.001
FINS (*μ*U/mL)	5.83 (4.20–9.28)	7.89 (5.90–9.91)	8.44 (5.86–11.25)	8.30 (5.94–11.48)	<0.001
2 h INS (*μ*U/mL)	27.62 (16.32–47.32)	33.52 (20.67–49.04)	39.60 (22.25–61.16)	36.55 (23.53–65.52)	0.002
HOMA-IR	1.27 (0.85–2.08)	1.78 (1.36–2.30)	1.93 (1.31–2.68)	2.08 (1.39–2.73)	<0.001
HbA_1_c (%)	5.40 ± 0.31	5.53 ± 0.39	5.57 ± 0.46	5.64 ± 1.13	0.060
TC (mmol/L)	4.80 (4.20–5.60)	5.05 (4.20–5.80)	5.00 (4.70–5.60)	5.30 (4.70–5.80)	0.013
LDL-c (mmol/L)	3.01 ± 0.86	3.16 ± 0.88	3.22 ± 0.83	3.28 ± 0.94	0.137
HDL-c (mmol/L)	1.36 ± 0.32	1.28 ± 0.26	1.27 ± 0.31	1.25 ± 0.30	0.035
TG (mmol/L)	1.00 (0.80–1.40)	1.15 (0.90–1.73)	1.40 (1.00–1.85)	1.60 (1.10–2.10)	<0.001

Variables with normal distributions are presented as mean ± SD; skewed variables are presented as the median value (interquartile range) [M (IQR)].

The chi-squared test was used for categorical values and ANOVA test for continuous values.

Abbreviations as in Table 1.

**Table 4:** Correlations between baseline fractalkine and metabolic parameters at year 2 (*n* = 459).

	Unadjusted		Age-, sex-, education-, smoking-, and drinking-adjusted	
	r	P value	*r*	*P* value
BMI	0.24	<0.001	0.23	<0.001
WC	0.28	<0.001	0.27	<0.001
WHR	0.24	<0.001	0.24	<0.001
Fat%	0.12	0.009	0.18	<0.001
SBP	0.11	0.054	0.04	0.470
DBP	0.11	0.053	0.07	0.187
FPG^a^	0.20	<0.001	0.16	0.001
2 h PG^a^	0.22	<0.001	0.20	<0.001
FINS^a^	0.20	<0.001	0.17	0.001
2 h insulin^a^	0.17	<0.001	0.21	<0.001
HOMA-IR^a^	0.23	<0.001	0.21	<0.001
HbA_1_c	0.10	0.037	0.11	0.024
TC^a^	0.14	0.011	0.10	0.058
LDL-c	0.11	0.033	0.08	0.130
HDL-c	−0.16	0.001	−0.18	<0.001
TG^a^	0.24	<0.001	0.23	<0.001

Abbreviations as in Table 1.

Coefficients were performed using Pearson's correlation analysis.

^a^lg(x) transformation was performed because of a skewed distribution

**Table 5:** Multiple logistic regression analysis of baseline fractalkine quartiles in the prediction of MetS and its components at year 2: ORs (*n* = 390).

ORs (95% CI)	Q1	Q2	Q3	Q4	*P* for trend
	≤0.28 ng/mL	0.28–0.43 ng/mL	0.43–0.64 ng/mL	>0.64 ng/mL	
MetS					
Model 1	1	1.61 (0.73–3.52)	2.81 (1.17–6.74)	7.05 (2.26–18.00)	<0.001
Model 2	1	1.64 (0.75–3.63)	2.89 (1.19–6.98)	7.18 (2.28–18.59)	<0.001
Model 3	1	1.80 (0.79–4.10)	2.67 (1.08–6.59)	5.31 (1.65–14.09)	0.016
Model 4	1	1.81 (0.79–4.16)	2.65 (1.06–6.61)	5.17 (1.60–13.74)	0.019
Model 5	1	1.67 (0.75–3.73)	2.86 (1.17–6.99)	5.73 (1.79–14.34)	0.007
Model 6	1	1.50 (0.66–3.40)	2.64 (1.06–6.56)	5.94 (1.85–15.09)	0.006
Central obesity					
Model 1	1	1.46 (0.75–2.84)	2.44 (1.19–4.99)	5.00 (2.17–11.50)	<0.001
Model 2	1	1.43 (0.73–2.80)	2.37 (1.15–4.87)	4.83 (2.09–11.19)	<0.001
Model 3	1	1.28 (0.62–2.65)	1.60 (0.73–3.48)	2.46 (0.99–6.03)	0.146
Model 4	1	1.46 (0.69–3.06)	1.51 (0.72–2.99)	2.18 (0.95–6.01)	0.126
Model 5	1	1.44 (0.73–2.84)	2.34 (1.13–4.84)	4.17 (1.78–9.79)	0.004
Model 6	1	1.41 (0.72–2.80)	2.36 (1.14–4.91)	4.58 (1.96–10.72)	0.001
Elevated BP					
Model 1	1	0.54 (0.29–1.11)	1.10 (0.59–2.05)	1.08 (0.59–2.01)	0.069
Model 2	1	0.53 (0.28–1.10)	1.09 (0.58–2.07)	1.03 (0.55–1.93)	0.078
Model 3	1	0.54 (0.28–1.12)	1.03 (0.54–1.97)	0.86 (0.45–1.64)	0.150
Model 4	1	0.49 (0.27–1.04)	0.94 (0.49–1.80)	0.77 (0.40–1.48)	0.118
Model 5	1	0.53 (0.28–1.11)	1.08 (0.57–2.04)	0.90 (0.47–1.72)	0.105
Model 6	1	0.52 (0.28–1.08)	1.05 (0.55–1.99)	0.95 (0.50–1.80)	0.085
Hyperglycemia					
Model 1	1	1.65 (0.82–3.32)	1.87 (0.92–3.79)	3.53 (1.60–7.81)	0.014
Model 2	1	1.66 (0.82–3.35)	1.89 (0.93–3.83)	3.61 (1.63–8.02)	0.013
Model 3	1	1.69 (0.83–3.43)	1.83 (0.90–3.74)	3.35 (1.49–7.55)	0.029
Model 4^a^	1	1.71 (0.84–3.51)	1.86 (0.90–3.84)	3.40 (1.49–7.75)	0.029
Model 5	1	1.68 (0.83–3.41)	1.84 (0.90–3.75)	3.07 (1.24–7.14)	0.071
Model 6	1	1.69 (0.83–3.43)	1.92 (0.94–3.92)	3.57 (1.60–7.99)	0.016
Hypertriglyceridemia^b^					
Model 1	1	1.37 (0.71–2.64)	1.82 (0.93–3.55)	2.69 (1.33–5.44)	0.038
Model 2	1	1.36 (0.70–2.62)	1.78 (0.91–3.50)	2.63 (1.30–5.34)	0.046
Model 3	1	1.38 (0.71–2.68)	1.72 (0.87–3.38)	2.28 (1.01–4.69)	0.141
Model 4	1	1.09 (0.55–2.17)	1.33 (0.65–2.69)	1.75 (0.83–3.71)	0.460
Model 5	1	1.36 (0.70–2.63)	1.77 (0.90–3.47)	2.33 (1.03–4.79)	0.113
Model 6	1	1.33 (0.67–2.63)	1.82 (0.91–3.64)	2.46 (1.19–5.11)	0.080
Low HDL-c^b^					
Model 1	1	0.84 (0.34–2.10)	0.93 (0.38–2.32)	1.51 (0.58–3.98)	0.595
Model 2	1	0.86 (0.34–2.15)	0.96 (0.38–2.38)	1.59 (0.60–4.24)	0.550
Model 3	1	0.86 (0.34–2.16)	0.95 (0.38–2.38)	1.50 (0.56–4.05)	0.665
Model 4	1	0.92 (0.37–2.34)	1.04 (0.41–2.64)	1.69 (0.61–4.63)	0.603
Model 5	1	0.85 (0.34–2.14)	0.96 (0.39–2.40)	1.64 (0.61–4.42)	0.532
Model 6	1	0.92 (0.37–2.34)	1.05 (0.42–2.64)	1.78 (0.66–4.80)	0.502

OR: odds ratio, CI: confidence interval.

Model 1: adjusted for age, sex.

Model 2: further adjusted for education attainment, smoking, alcohol, and drinking based on model 1.

Model 3: further adjusted for visceral fat based on model 2.

Model 4: further adjusted for CRP based on model 3.

Model 5: further adjusted for HOMA-IR based on model 2; ^a^for hyperglycemia: further adjusted for FINS based on model 2.

Model 6: further adjusted for TG and HDL-c based on model 2.

^b^Not adjusted for itself.

Abbreviations as in Table 1.
