# Supplementary material for: Circulating Fractalkine Levels Predict the Development of the Metabolic Syndrome
Source: Int J Endocrinol. 2014 Apr 30;2014:715148. doi: 10.1155/2014/715148 (PMC4021752; doi:10.1155/2014/715148)
Supplement: Supplementary file 1 — Supplementary table 1. Baseline Characteristics of Participants Completely Investigated According to the Presence or Absence of the Mets (n=459). [file 715148.f1.pdf]

Supplementary table 1. Baseline Characteristics of Participants Completely Investigated According to the Presence or Absence of the Mets (n=459)

Variables	Total	Metabolic syndrome absent	Metabolic syndrome	P for tren[illegible]
n (case/control)	459	399	60	
Fractalkine(ng/ml)	0.43 (0.28-0.64)	0.40 (0.25-0.60)	0.65 (0.45-0.71)	<0.001
Age (years)	57.25±6.60	57.07±6.55	58.30±6.78	0.161
Male, n(%)	168 (36.6)	131 (32.8)	37 (61.7)	<0.001
Education level, n(%)				0.803
Less than high school	47 (10.2)	41 (10.3)	6 (10.0)	
High school	348 (75.8)	303 (75.9)	45 (75.0)	
More than high school	64 (13.9)	55 (13.8)	9 (15.0)	
Current smoker,n(%)	359 (78.2)	322 (80.7)	37 (61.7)	0.001
Alcohol drinker,n(%)	365 (79.5)	325 (81.5)	40 (66.7)	0.008
BMI(kg/m^2)	23.43±2.81	23.02±2.65	25.83±2.50	<0.001
WC (cm)	78.18±9.02	76.51±8.27	88.00±6.69	<0.001
WHR	0.87±0.07	0.85±0.07	0.94±0.05	<0.001
Fat% (%)	29.29±6.80	28.83±6.69	31.99±6.84	<0.001
SBP (mm Hg)	124.31±16.68	122.32±15.94	135.91±16.23	<0.001
DBP (mm Hg)	81.45±9.92	80.39±9.71	87.61±8.88	<0.001
FPG (mmol/L)	4.89 (4.50-5.22)	4.83 (4.50-5.17)	5.11 (4.83-5.94)	<0.001
2h PG (mmol/L)	5.50 (4.50-6.67)	5.33 (4.33-6.33)	7.22 (5.28-10.17)	<0.001
FINS (μU/ml)	11.09 (8.23-14.09)	10.61 (8.11-13.32)	14.95 (11.98-18.54)	<0.001
2h INS (μU/ml)	58.92 (37.91-97.05)	55.68 (35.96-82.56)	86.13 (52.29-136.56)	<0.001

HOMA-IR	2.38 (1.79-3.20)	2.25 (1.71-2.99)	3.44 (2.60-5.28)	<0.001
$HbA1_C$ (%)	5.67±0.65	5.62±0.60	5.91±0.84	0.001
TC (mmol/L)	5.57 (4.90-6.22)	5.52 (4.87-6.19)	5.36 (4.25-8.00)	0.070
LDL-c (mmol/L)	2.40±0.57	2.40±0.56	2.41±0.62	0.971
HDL-c (mmol/L)	1.46±0.37	1.52±0.35	1.11±0.23	<0.001
TG (mmol/L)	1.30 (0.95-1.84)	1.23 (0.88-1.58)	2.51 (2.29-2.84)	<0.001
CRP (mg/dL)	0.62 (0.30-1.67)	0.58 (0.30-1.65)	1.19 (0.72-3.49)	0.021
SFA (cm^2)	156.50 (119.05-205.03)	155.25 (116.38-205.00)	163.40 (126.73-207.58)	0.027
VFA (cm^2)	68.96 (46.07-109.25)	63.92 (41.40-97.49)	117.45 (85.79-167.38)	<0.001
Central obesity (%)	55 (11.98)	26 (6.5)	29 (48.3)	<0.001
Elevated BP (%)	242 (52.7)	186 (46.6)	56 (93.3)	<0.001
Hyperglycemia (%)	75 (16.3)	44 (11.0)	31 (51.7)	<0.001
Hypertriglyceridemia (%)	137 (29.8)	80 (20.1)	57 (95.0)	<0.001
Low HDL-c (%)	41 (8.9)	11 (2.8)	30 (50.0)	<0.001

Variables with normal distributions are presented as mean ± SD; skewed variables are presented as the median value (inter-quartile range) [M (IQR)]. The Chi-squared test was used for categorical values and t-test for continuous. P for trend depicts the significance in the difference of the mean values between participants with and without metabolic syndrome.

Abbreviations as in Table 1.
